# Effective Veterinary Clinical Teaching in a Variety of Teaching Settings

**DOI:** 10.3390/vetsci9010017

**Published:** 2022-01-05

**Authors:** Amanda Nichole (Mandi) Carr, Roy Neville Kirkwood, Kiro Risto Petrovski

**Affiliations:** 1 Davies Livestock Research Centre, School of Animal and Veterinary Sciences, The University of Adelaide, Roseworthy, SA 5371, Australia; mandi.carr@adelaide.edu.au; 2 School of Animal and Veterinary Sciences, The University of Adelaide, Roseworthy, SA 5371, Australia; roy.kirkwood@adelaide.edu.au; 3 Australian Centre for Antimicrobial Resistance Ecology, School of Animal and Veterinary Sciences, The University of Adelaide, Roseworthy, SA 5371, Australia

**Keywords:** animal science, clinical activities, clinical practice, teaching, traditional academic setting, veterinary learners, work-based learning

## Abstract

This review explores different modalities for clinical teaching of veterinary learners globally. Effective clinical teaching aims to prepare graduates for a successful career in clinical practice. Unfortunately, there is scant literature concerning clinical teaching in veterinary medicine. Our intent for this review is to stimulate and/or facilitate discussion and/or research in this important area. We discuss the different forms that veterinary clinical teaching can take, depending on their setting, which can be university-based clinical activities, work-based in commercial clinical practices, or in a traditional academic setting with little to no real-time exposure to clients and patients. We suggest that each of these modalities has a place in clinical teaching of veterinary learners at any point in the curriculum but that a mix of these approaches will likely provide an improved experience for the learner. Further, we discuss strategies to improve clinical teaching in these different settings. Potential strategies related to the teaching skills of clinical instructors could include training in delivery of clinical teaching in a variety of learning settings, and instructors’ official recognition, including opportunities for career progression. Potential strategies to improve clinical teaching in different teaching settings would vary with the learning settings. For example, in traditional academic settings, case-based learning with incorporation of simulation models is one proposed strategy. The involvement of learners in ‘teach-others’ is a strategy for both traditional academic and clinical settings. Finally, clearly addressing Day One competencies is required in any clinical teaching setting.

## 1. Introduction

**Figure 1:**
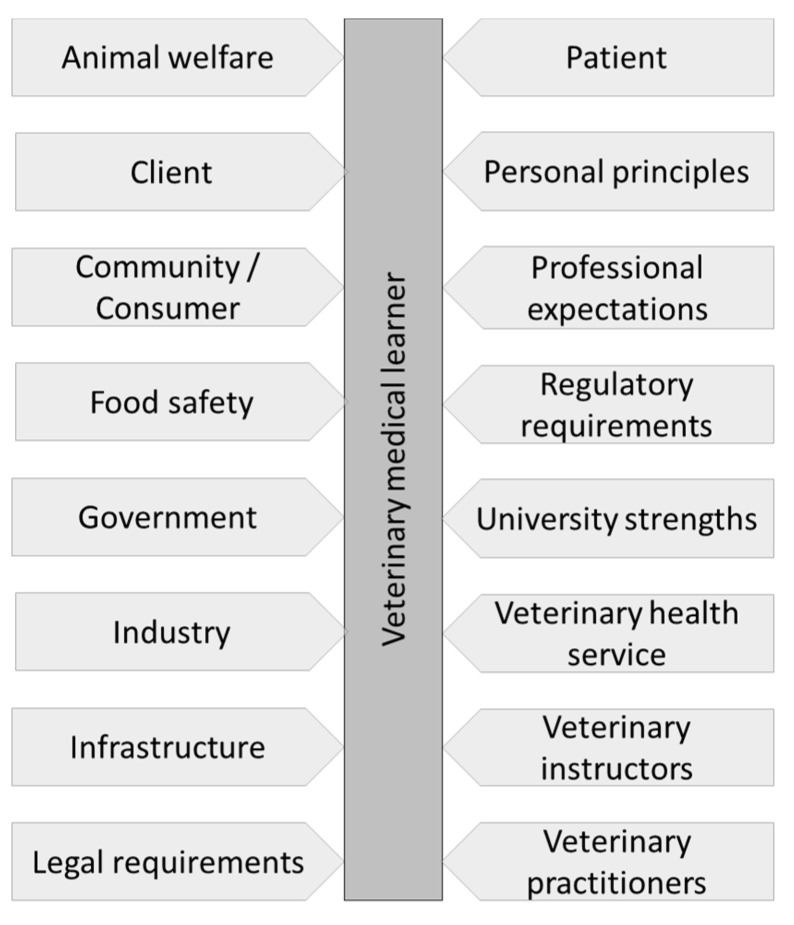
Factors to consider in veterinary clinical education and practice.

The aim of clinical teaching in veterinary medicine is preparing graduates to meet all required veterinary graduate attributes. As illustrated in Figure 1, there are many factors to consider when designing or assessing veterinary clinical education. The veterinary learner needs to understand the various potential constraints in their clinical education. In addition to government and professional regulations, the animal and their welfare are essential considerations, as is the impact of food animal clinical practice on food safety. Indeed, the environmental considerations around repercussions of veterinary products (e.g., diclofenac and the Asian Vulture Crisis) parallel food safety and welfare [1,2].

One of the cornerstones in the development of veterinary learners and their transition into practitioners is the exposure to practice. For achievement of the learning objectives related to exposure to practice, the clinical teaching requires commitment by all involved parties: the staff in the clinical environment, learners, instructors, and involved clients/patients. Exposure to practice (experiential learning) is aimed at assisting veterinary learners to develop veterinary medical and professional attributes within the specific clinical context of the work. Typically, experiential learning is delivered by rotations through a variety of clinical settings and sub-specialties, either at university or community veterinary service providers [3,4,5]. Some universities have developed university-based primary practices, e.g., in collaboration with technical high schools [6]. Experiential learning allows learners to advance their clinical reasoning and technical skills, communication, deepen their appreciation of practice management, and work within economic constraints whilst providing optimal care for the client/patient [6,7,8,9]. Learner confidence and their capacity to apply experiences in new learning settings are improved [8,10]. Much of the experiential learning is delivered on an ‘apprenticeship’ model basis, where learning occurs during the clinical encounter, in contact with the client and the patient [5]. Clinical teaching methodology should not be restricted to the ‘clinical years’ but rather be addressed throughout the curriculum, as we have discussed previously [11]. Veterinary school accreditation bodies and some registration authorities tightly regulate the minimum requirements of experiential learning of veterinary learners. Many of these requirements are historic, before animal welfare standards and other ethical responsibilities were set. Over time, a number of factors have changed the learning environment (Box 1) and now alternative approaches to delivering some of the experiential learning may need to be considered.

Box 1 **Factors that have led to the need for alternative methods of delivery of clinical teaching in lieu of exposure to real-life practice. AAVMC—American Association of Veterinary Medical Colleges (North America); EAEVE—European Association of Establishments for Veterinary Education (Europe); RCVS—Royal College of Veterinary Surgeons (United Kingdom); VSCAAC—Veterinary Schools Accreditation and Advisory Committee (Australasia).**

- Accreditation/Regulatory Requirements (e.g., AAVMC, EAEVE, RCVS, VSAAC)
- Alternative approaches for learners refusing clinical teaching with live animals, which is a growing concern
- Animal rights activism
- Availability of animal models in Lab skills in order to avoid the use of live animals
- Caseload per clinical instructor
- Demand for exposure to different settings
- Departmental pressure
- Following the example from other areas of the OneHealth initiative
- Increasing learner numbers
- Increasing specialization at university veterinary hospitals
- Interruptions to delivery (e.g., Covid-19 pandemic)
- Minimal access to live animals, including restrictions on availability of shelter animals
- Staff shortages

Cited literature [3,4,5,12,13,14,15,16]

One of the considerations should be the involvement of community-based veterinary service providers in clinical teaching of veterinary learners, both for clinical setting and work-based learning. Many veterinary schools have taken advantage of these opportunities with some having completely dispersed their clinical teaching off site, whilst others have restricted such exposure only to work-related learning. Independently of the approach to the experiential learning delivery, it is important to maintain the integrity of training within prescribed ethical limits.

Unfortunately, despite a significant proportion of veterinary medical education occurring in clinical settings, the literature describing teaching approaches during exposure to practice are limited. Therefore, we felt that this review could facilitate discussion and/or research in this area. 

Clinical teaching can be delivered in a variety of settings [3,11,16,17,18] with successful clinical teaching depending on a variety of factors, including the complex interplay between personalities, beliefs and teaching settings [17,19]. Achieving the learning objectives and attaining the graduate attributes of veterinary learners are less affected by the setting where the clinical encounter occurs but more important are the skills of the clinical instructor to deliver the clinical teaching using a multidimensional approach [12,17]. Additionally, clinical teaching can provide the connection between universities and veterinary health providers [5].

Finally, the importance of mental health support in veterinary practice should not be forgotten. Veterinary learners/practitioners are three times more likely to commit suicide compared to the general population [20]. Clinical teaching could play a vital role in legitimizing that veterinary practice can take various emotional tolls, which also translate to physical repercussions [20,21].

In this review, enhanced by the experience of authors from a number of workshops on clinical teaching, we will describe

- Main differences between clinical teaching in three types of learning settings, (clinical, traditional academic, and work-based learning) related to the delivery of the ‘exposure to practice’;
- Main differences between cases seen/solved in the three types of learning settings
- Structuring clinical teaching for high efficacy;
- Potential clinical teaching methods to minimize the impact of the types of learning setting on learning outcomes.

## 2. Main Differences between Clinical Teaching Settings

Teaching settings will influence the clinical experience of learners, their assessments and learning outcomes, as well as the responsibilities of the involved parties. Different teaching settings include clinical settings where clinical activities occur in a university setting, traditional academic settings with little or no real-time animal contact, and work-based learning settings whereby learners are placed in a commercial practice. Indeed, even in traditional academic settings, use of live animals is provided (e.g., practical activities) but animal welfare often precludes repetition until mastery of a particular competence. Each setting has their advantages and disadvantages. The clinical setting gives case exposure under controlled conditions but may require ethics approval while the traditional academic setting does not require animals so benefits from the lack of approvals required and delivery in ethically justified conditions. Ethics approval may be required for clinical settings for any elective procedures. As there are no ethical considerations, the activity can be repeated until the learner achieves mastery in the specific attribute/skill. The work-based setting is ‘real world’ but will require limitations on learner involvement (Table 1).

## 3. Main Differences between Cases Seen/Solved

The teaching setting will dictate the approach to clinical teaching, with impacts on both instructor and learner involvement and responsibilities. The type of cases seen will also be a function of the clinical teaching setting (Table 2). In (university) clinical settings many clinical encounters are referrals, but primary cases are also common in some clinics. Learners may see only a selection of these and may be allowed to conductresearch with an aim to developing a deeper understanding of the case. In traditional academic settings, the learner may be provided with a case-based scenario and, usually working in a group, solves the case. Both settings have close supervision and opportunities for discussion. In work-based learning settings, cases are variable, and learner may be exposed to more cases but may have less opportunity to develop deep understanding of the cases and the supervision is less intensive.

## 4. Structuring Clinical Teaching for High Efficacy

Clinical teaching should be structured in a way that ensures effective teaching and stimulates deep-learning [10,12,27,28,29] (Table 3). The number of cases seen appears to be less important than the opportunity to be involved [3,14,17,30]. A real involvement of the learner would provide opportunity for development of clinical reasoning skills. There must be opportunities to develop deep learning [30] which may be achieved by traditional delivery or blended delivery of the clinical teaching [31,32,33]. The opportunity to teach others should be utilized [30,34]. This should be independent of the type of case (e.g., case-based scenario or a ‘real’ clinical case, either carefully selected or ‘whatever comes through the door’ (the ‘clinical encounter’). For stimulation of deeper learning, the management of the case encounter can utilize a clinical teaching model, such as the Five microskills model, including debriefing with elements of reflection [11].

## 5. Potential Strategies to Minimize the Impact of Alternative Settings and Approaches on Learner Outcomes

The main perceived deficit of the alternatives to exposure to real clients and patients is the lack of a ‘clinical encounter’. To minimize the potential negative impact of alternative approaches to exposure to practice, these must be viewed as complimentary rather than being relied on as a single approach to delivery [35]. For any of the proposed strategies to be effective, it is important they be clearly elaborated in the orientation of learners. In that way, learners are aware of the process, expectations and responsibilities. Strategies to minimize the impact of alternative approaches on the learner’s experience and training during the curriculum must address the efficacy of the clinical teaching through addressing

- Clinical teaching skills of instructors;
- Teaching settings;
- Time spent per site, and in total, on experiential learning.

### 5.1. Clinical Teaching Skills of Instructors

- **Training of instructors in both delivery of clinical teaching and pedagogy-based approaches.** Trained clinical instructors are better at delivering clinical teaching and are usually more satisfied [4,15,40,41,42]. Currently, veterinary instructors rarely, if ever, receive official training in clinical teaching, and there are no standardized requirements. This has been identified as an important gap in both veterinary [43,44] and human medical education [15,41,44,45,46,47]. Veterinary schools should provide opportunities to all professional staff for training in education, particularly staff delivering clinical teaching. In some geographical areas, on-line courses in general education become attractive [47,48]. However, it is recognized that general training may not be attractive or satisfying for veterinary clinical educators. Discipline teaching is preferred [47,49]. Indeed, some schools have developed discipline-specific, context-relevant partial programs to complete the clinical instructor development program [49], including the school at which we teach.
- **Official recognition of clinical instructors**. Official recognition of clinical instructors may take the form of being given title-holder status or, even better, a special qualification as a clinical instructor, after completing a specified training course/module/curriculum [35]. Training can be managed at departmental level, and it is easy to achieve.
- **Promotion and career advancement of clinical instructors**. The possibility of promotion of clinical instructors within a university setting is limited [16,41,50]. This is often due to the lack of a research component by many of the clinical instructors [51,52,53]. Promotion is primarily managed at the university, not departmental, level using the same rigid requirements as traditional academics. This makes the recognition by promotion and career advancements of clinical instructors more difficult and limited [50]. Therefore, for promotion and career advancement of clinical instructors, universities must consider having a special track for clinical instructors in veterinary medicine. Indeed, career progression is important also in partner clinics and this is an important area that needs to be addressed by the industry.

### 5.2. Strategies to Improve Clinical Teaching in Different Teaching Settings

#### 5.2.1. Traditional Academic Settings

Even the best approach to clinical teaching, in isolation, cannot replace clinical exposure. The difference between doing things and trying to simulate all that may happen during a clinical encounter should be considered. Hence, traditional academic settings should be utilized to facilitate development of clinical reasoning skills in conjunction with clinical exposure [16,54].

**Figure 2:**
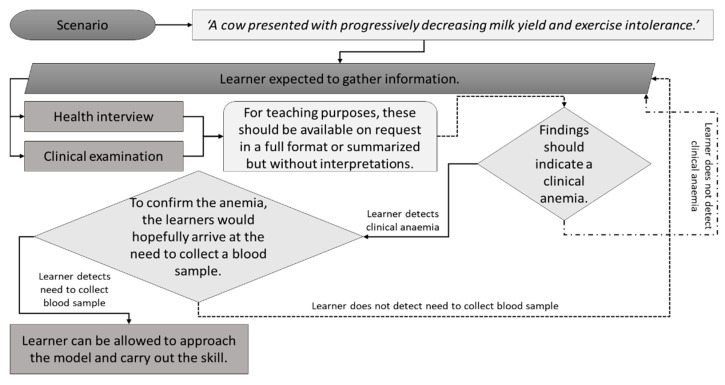
An example of incorporation of a simulation model within a case-based scenario rather than a practical class only using a decision-making algorithm.

**Figure 3:**
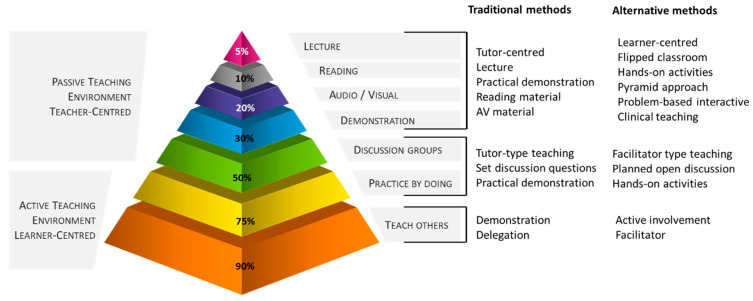
The effect of teaching method on the retention of knowledge by learners demonstrating the need of a component of ‘teach others’ by learners in the clinical teaching. Adapted from [56]. AV—Audio-visual.

- **Case-based learning (CBL)**. The CBL is a clinical teaching method usually applicable for earlier stages of the learner’s development. However, skillfully used, it is also suitable in advanced learning. The advantage of the CBL is it is a less time-consuming approach to delivery of clinical teaching. The role of the clinical instructor, who is the ‘expert in the field’ is to correct, direct, and provide feedback [55].
  ○ **Incorporation of simulation models within a case-based scenario rather than a practical class only,** e.g., when using a bovine venipuncture simulation model, the scenario would look like: *“A cow presented with progressively decreasing milk yield and exercise intolerance”.* The scenario should guide learners to gather additional information
    ■ Obtain health interview information from the client (for teaching purposes it should be available on request in a full format or summarized but without interpretations);
    ■ Clinical examination results of the patient (for teaching purposes it should be available on request in a full format or summarized but without interpretations);
    ■ Findings should indicate a clinical anemia. To confirm the anemia, the learners would hopefully arrive at the need to collect a blood sample;
    ■ At that moment learners can be allowed to approach the model and carry out the skill;
    ■ These points are summarized in Figure 2.

- **Day One competencies (graduate attributes) addressed.** Learners prefer seeing practice that is closer to what they will be seeing in their future clinical practice. Therefore, experiential learning should include diverse cases, particularly those that are common in general practice [13,19]. Learners prefer community-based clinical encounters, but with this approach only, there is often a lack of continuity of care. Thus, a variety of teaching settings are the preferred option.
- **Elements of ‘teach-others’.** ‘Teaching’ others significantly increased knowledge retention and capacity to implement experience from one-to-another clinical situation in the future [30,34,56] (Figure 3). Teach-others learning may be used during discussion with the client, when implementing peer-assisted learning [14], or utilizing mini-lecture discussions [30]. Indeed, peer-assisted learning may also be a valuable tool for feedback on performance, frequently accepted as less confronting than when provided by the clinical instructor [14,31,55]. It is worth noting that peer-based feedback only is not preferred, as learners appreciate expert opinion on their performance and progress. Hence, a combination of peer- and expert-feedback is needed.

- **Psychomotor activity within the case-solving.** For example, use of simulation models rather than only presenting a case for solving.
  ○ Simulation in a variety of forms should be utilized as much as possible, including but not restricted to [57,58,59,60,61]:
    ■ High-fidelity examination simulators;
    ■ Single-task models (e.g., skin suturing model, model for venipuncture);
    ■ Standardized patient encounters;
    ■ Virtual reality models (e.g., ‘haptic cow/horse’).
- **Team-based learning (TBL)**. Although TBL is a teaching method aimed at delivery of clinical teaching to early stages of development of learners in veterinary and medical sciences, it is assumed to be very suitable for clinical teaching in advanced years [32,55,62]. Team-based learning clinical teaching has been praised for three elements of clinical teaching, inclusion of development of clinical reasoning, team work from learner’s perspective [31], and time efficiency. It usually consists of in-contact activities of 2 h duration, but preparation time is required. For efficacy, TBL should be properly executed, namely learners should be carefully allocated to groups. There should be a proper pre-activity preparation (e.g., readings, pre-recorded lectures) and individual- and group-level testing should be incorporated (i.e., readiness assurance tests), followed by immediate feedback on performance. Use of the four S approach (significant problem, same problem, specific choice and simultaneous reporting) during the activity is relevant for reflection, clinical reasoning and problem-solving [31,55,62,63].
- **Combination of any of the above proposed strategies.**

#### 5.2.2. Clinical Setting

Learners do not always recognize every teaching effort in clinical settings [12,16]. Usually, they do not recognize the utility of other means of exposure to clinical encounters not directly involving client/patient discussion [16] (e.g., journal clubs, seminars, tutorials). Therefore, guidance should be provided regarding strategic utility when using alternate strategies to improve clinical teaching.

- Elements of ‘teach-others’.
- Clinical cases that are relevant to what the learners will be seeing in practice [3,19].
- Case-based learning (e.g., rounds presentation/s). Case based discussions and enquiries are a great learning opportunity for learners [19]. However, this activity should not be limited to presentation and interpretation of facts only. It should stimulate development of clinical reasoning and other analytical skills [54,64]. For stimulation of the deep learning in learners, provided the learner has been asked to present rounds, the presentation should consider a type of a case critique where the learner explains what, why and how it could be improved in the particular clinical encounter.
- Day One competencies (graduate attributes) addressed. Minimize administrative load of learners [3].
- Student-lead clinic. These have been reported to increase the deep learning approach to clinical encounters by learners [19,65].
- Use of clinical teaching models such as the Five Microskills Model. The use of clinical teaching models stimulates discussion and enquiries related to the clinical encounter that are particularly useful in the development of clinical reasoning of learners, yet with minimal time requirements of the clinical instructor in a busy practice [11,19].
- Using clinical instructor/s with a relevant clinical experience (e.g., avoiding the use of interns) [3].

#### 5.2.3. Work-Based Learning

Work-based learning may be perceived as forced and not always according to the temporary aspirations of the learners. However, within the current environment, regulatory authorities still require omni-competency and work-based learning needs to incorporate a diverse experiential learning. Some schools aim to use the work-based learning to attract learner’s attention to areas that are currently experiencing a shortage of practitioners [66]. The biggest issues with this approach to clinical teaching are the limited time spent with the client/patient, lack of continuous feedback, and variability in clinical encounters by type and numbers [19]. Additionally, as most of the clinical teaching in work-based learning is only observational, anecdotal evidence from our learners is that there is a desire for them to have more active involvement.

- **Involvement in the clinical encounter**. Learners may be involved by brief teaching models (e.g., Five Microskills [11]) or a formative assessments (e.g., grand rounds presentation/s).
- **Day One competencies (graduate attributes) addressed** [13].
- **Portfolio-based learning** can be utilized to stimulate the learner to engage with the clinical exposure [64]. However, for a deep learning, at least part of the records in the portfolio should include elements of displaying clinical reasoning and other analytical skills [64]. A properly designed portfolio should also include some elements of reflection [64]. Alternatives to a full learning portfolio, encompassing only particular portion/s, may be more appropriate and less demanding on the learner and the clinical instructor.

### 5.3. Strategies to Improve Clinical Teaching Changing the Time Spent in Exposure to Practice

Increased weeks of exposure to practice within a specified teaching setting has been identified as a possibility to enhance a learner’s experience [3,35]. In veterinary medicine, the number of rotations (usually animal species and some discipline-related) dictates a number of experiential learning sites. Therefore, increasing the length of exposure to experiential learning sites would ultimately require extension of the clinical part of the curriculum.

## 6. Conclusions

From the literature reviewed, we conclude that effective veterinary clinical teaching of graduates is central to post graduate clinical success. We have presented various clinical settings and strategies affecting clinical teaching and learning and recommend that, as far as possible, the employment of a mixture of strategies (blended delivery) will enhance student satisfaction and clinical success. Indeed, delivery of clinical teaching must be cognizant of animal welfare requirements and the 3 Rs (Reduction, Refinement and Replacement) of using animals in research and teaching [67]. We have proposed some strategies for clinical teaching but learner satisfaction and employer opinion, as well as instructor and learner wellbeing with the incorporation of a blended clinical teaching approach, are yet to be measured (e.g., using anonymous on-line surveys or targeted interviews).

## Figures and Tables

**Table 1:** Differences between clinical teaching settings on learner assessment and learning outcomes. Bolded text—difference between parties.

Parameter	Clinical Settings	Traditional Academic Settings	Work-Based Learning
Assessment method	Assessment of competency standards, Direct observations, Review of documentation	Assignment, Orals, OSCE ^1^, Project, Written	Direct observations; Semi-formative
	Continuous and cumulative	Episodic	Hopefully continuous and cumulative
Consequences of mistakes	Adverse effects on future learning and risk-taking, Adverse effects on the patient and/or client, Failure, Loss of employment opportunities, Loss of self-esteem, Poor grades	Failure, Lost opportunities for a scholarship and early graduation, Poor grades	Adverse effects on future learning and risk-taking, Adverse effects on the patient and/or client, Loss of employment opportunities, Loss of self-esteem, Repeating the activity
Emphasis of the learning material	Integration of skills, knowledge and attributes; Involvement of an integrated approach to all three learning domains (cognitive, affective and psychomotor)	Hopefully involves all three learning domains (cognitive, affective and psychomotor) but often in isolation, Knowledge, Theory, Skills;	Integration of cognitive and affective skills ± psychomotor skills
Expected outcomes	Each party may have different expectations and concerns. **Learner and instructor**—Achievement of academic program requirements, Good patient outcome, Happy client **Client**—Economic return, Improved animal welfare, Recovery of patient	Achievement of academic program requirements	Achievements of the work-based learning objectives
Group size	Usually single to few learners	Usually few to many learners	Usually single to few learners
Instructor’s responsibilities	Available; Colleague; Counsellor; Demonstrator; Evaluator, Facilitator, Mentor, More personal, Objective, Prepared, Role model, Up to date	Evaluator, Facilitator, Mentor, Less personal, Objective, Prepared, Up to date, Well presented, Work within specified frame	Colleague, Demonstrator, Evaluator, Facilitator, Mentor, More personal Prepared, Up to date, Objective, Role model, ±Available, ±Counsellor
Involved parties	Academic supervision, Client, Client’s family, Clinical and Para-clinical staff, Enterprise employees, Institutional supervision, Instructors, Learners, Patient	Instructors, Learners, Librarians, Para-teaching staff, Simulated clients	Academic supervision, Client, and Client’s family, Clinical and Para-clinical staff, Enterprise employees, Institutional supervision, Instructors, Learners, Patient
Learner’s responsibilities	Demonstrate problem-solving, clinical reasoning and empathy, Demonstrate professionalism, safe and effective clinical practice,	Participate, Pass assessment tasks, Turn up to activities, Work cooperatively with peers in group activities and assessments	Demonstrate professionalism, Turn up to activity, Work cooperatively
Learning happens by	Engagement, Learning through experience and active involvement, Observation, On-going feedback, Peer interaction, Real-life practice, Trial and error	Group work, Learner-centered strategies, Occasional feedback, Reading, Problem-based activities, Simulated practice	Learning through experience, Observation; Occasional feedback
Learning setting	Client’s homes or properties, Clinic, Hospital, Other institutions, Private institutions	Animal handling facility, Classroom, Laboratory, Tutorial room	Client’s homes or properties, Clinic, Hospital, Other institutions, Private institutions
Main settings	Clinical activities occur in a university clinic or similar setting	Little or no real-time animal contact (mainly theoretical, except practical activities)	Learners placed in a commercial practice
Number of instructors involved in clinical teaching	Usually few to many instructors	Usually single to few instructors	Usually single to few instructors
Risk of conflict	High	Low	Low to medium
Time per clinical encounter	Short to medium	Medium to long	Short
Total learner-instructor contact time	Short to medium	Medium to long	Short

^1^ Objective, structured clinical examination. Cited literature [9,11,22,23,24,25,26].

**Table 2:** Influence of clinical teaching setting on instructor and learner involvement and responsibilities for clinical encounters.

Parameter	Clinical Settings	Traditional Academic Settings	Work-Based Learning
Instructor’s involvement	Facilitate learner’s dealing with cases; Learner’s assessment	Facilitate case-solving by learners; Learner’s assessment; Provision of feedback	Deal with cases and allow learners to observe ± be involved
Instructor’s responsibilities	Create safe learning environment during clinical exposure	Present for solving of a case in safe learning environment	Expose learners to cases
Learner’s involvement	Have the role of an ‘intern’ under direct and immediate supervision	Case-solving under direct facilitation	Minimal to restricted
Learner’s responsibilities	Deal with cases and observe clinic operations; Prepare case notes	Participate in case-solving	Observe cases and clinic operations
Learning environment	Ensuring achievement of learning objectives; ‘Safe’	Animal handling facility; Classroom; Laboratory; Tutorial room; ± Simulated house call/field environment	Regular clinical practice
Learning objectives	Set learning objectives	Instructor-led set learning objectives	Prepared by learner and agreed by instructor (principles of self-directed learning)
Type of cases	Few; Need research time, Selection of what comes ‘in the door’, Unpredictable	Few; Clearly selected by the instructor, Predictable, Simulations	Anything that comes ‘in the door’; As many as possible, Unpredictable

**Table 3:** Structuring the clinical teaching for high efficacy.

Strategy	Attributes
Clinical encounter	Case-based/Real/Simulated; Carefully selected to allow progression of the learner from ‘simple’ to ‘more complex’; Stimulate teamwork
Independent learning	Case of the day/week; Case/Exit/Grand rounds; Question banks; Poster; Project; Report
Orientation	Discuss the usual approaches; Find out learners’ expectations/level of knowledge and expertise; Introduce facility; Introduce team; Set time for feedback; Set expectations; Set levels of responsibility
Planning	Debriefing after dealing with client/patient; Levels of responsibility when dealing with client/patient; How/What/When/Where/Who/Why when dealing with client/patient; Responsibility for veterinary medical records
Reflection	Debriefing on every case; What went well; What could be improved

Cited literature [12,35,36,37,38,39].

## Glossary

Case-based learning	solving of an authentic clinical case using clinical reasoning skills, particularly useful in developing learners’ reflection and analytical skills through peer-learning approach and activation of prior knowledge. Learner-centered approach to learning.
Clinical encounter	any physical or virtual contact with a veterinary patient and client (e.g., owner, employee of an enterprise) with a primary responsibility to carry out clinical assessment or activity.
Clinical instructor	in addition to the regular veterinary practitioner’s duties, a clinical instructor should fulfil roles of assessor, facilitator, mentor, preceptor, role-model, supervisor, and teacher of veterinary learners in a clinical teaching environment. Apprentice/intern in the upper years, Resident, Veterinary educator/teacher, Veterinary practitioner.
Clinical reasoning	process during which a learner collects information, process it, comes to an understanding of the problem presented during a clinical encounter, and prepares a management plan, followed by evaluation of the outcome and self-reflection. Common synonyms include clinical acumen, clinical critical thinking, clinical decision-making, clinical judgment, clinical problem-solving, and clinical rationale.
Clinical teaching	form of an interpersonal communication between a clinical instructor and a learner that involves a physical or virtual clinical encounter.
Deep learning	aiming for mastery of essential academic content; thinking critically and solving complex problems; working collaboratively and communicating effectively; having an academic mindset; and being empowered through self-directed learning.
Portfolio-based learning	record of examples of learner’s work, including but not limited to case log, activity log and similar, with some elements of demonstration of reflective and clinical reasoning skills used for learning and assessment purposes. Learner-centered approach to learning.
Proper learning or a safe learning environment	an environment in which a learner feels safe, relaxed, and willing to take risks in pursuing a goal; enhances self-esteem and encourages exploration.
Self-directed learning	learners take charge of their own learning process by identifying learning needs, goals, and strategies and evaluating learning performances and outcomes. Learner-centered approach to learning.
Team-based learning	solving of an authentic clinical case using clinical reasoning skills. Particularly useful in developing basic science concepts through peer-learning approach (learning occurs within a team but also between teams when activity carried out concurrently with more than one team) and activation of prior knowledge. Learner-centered approach to learning.
Work-based learning	educational method that immerses the learners in the workplace. Usually, learners have to complete typical tasks for the workplace and satisfy school accreditation requirements, e.g., the American Association of Veterinary Medical Colleges and The Royal College of Veterinary Surgeons (where applicable).
